# Neuromolecular Underpinnings of Negative Cognitive Bias in Depression

**DOI:** 10.3390/cells10113157

**Published:** 2021-11-13

**Authors:** Karolina Noworyta, Agata Cieslik, Rafal Rygula

**Affiliations:** Affective Cognitive Neuroscience Laboratory, Department of Pharmacology, Maj Institute of Pharmacology Polish Academy of Sciences, Smetna Street 12, 31-343 Krakow, Poland; k.a.noworyta@gmail.com (K.N.); agatacieslik1@gmail.com (A.C.)

**Keywords:** depression, cognitive, dopamine, serotonin, noradrenaline, judgment bias, feedback sensitivity

## Abstract

This selective review aims to summarize the recent advances in understanding the neuromolecular underpinnings of biased cognition in depressive disorder. We begin by considering the cognitive correlates of depressed mood and the key brain systems implicated in its development. We then review the core findings across two domains of biased cognitive function in depression: pessimistic judgment bias and abnormal response to negative feedback. In considering their underlying substrates, we focus on the neurochemical mechanisms identified by genetic, molecular and pharmacological challenge studies. We conclude by discussing experimental approaches to the treatment of depression, which are derived largely from an improved understanding of its cognitive substrates.

## 1. Introduction

Depression is the leading cause of disability in the 21st century, affecting an estimated 350 million people worldwide [1]. The number of people prescribed antidepressant medications, the first-line treatment for depressive disorder, increases each year, and the market for these medications is valued at billions of dollars [2]. However, the prevalence of depression has not decreased since accurate record-keeping began. One reason for this paradox is the failure of science to find a compelling biomedical explanation for depression and adequately address how and why this devastating condition occurs.

One of the most important yet still underappreciated symptoms of depression is aberrant cognition. Indeed, cognitive problems have been included in the diagnostic criteria for mood disorders, and according to the Diagnostic and Statistical Manual (DSM 5), the specific criteria for depression include a reduced ability to concentrate, disturbed memory and indecisiveness [3]. These cognitive problems are usually persistent, recurrent and significantly disrupt quality of life [4].

The symptomatology of depression suggests a processing bias toward negative aspects of the environment. Indeed, depressed individuals are more likely to recall negative autobiographical memories, and when they do recall positive experiences, they are overly general and lacking in detail [5]. Individuals also exhibit impaired recognition of happy facial expressions [6] and respond more rapidly to sad versus happy word targets [7]. The two most important characteristics of negatively biased cognition include pessimistic judgment bias [8,9] and catastrophic reactions to negative feedback [10,11].

Among the brain regions that have been most commonly implicated in the biased cognition associated with depression are the ventromedial (vlPFC) and orbitofrontal (OFC) cortices [12]. It has been suggested that the vlPFC and adjacent OFC are critical for making an association between a reinforced outcome and a given action, as well as for behavioral guidance once the expectancy of an outcome is violated [13]. A study by Wheeler and Fellows (2008) demonstrated that OFC is critical for learning from negative feedback [14]. Other important neuroanatomical correlates of negative processing bias include the dorsal and ventral striatum, the anterior insula extending to the lateral OFC and the cingulate cortex [12]. It has been suggested that the dorsal regions of the striatum are involved in forming habitual action selection following positive feedback, while the ventral regions mediate learning the value of positive feedback [15]. The insula has been suggested to be involved in the evaluation of negative outcomes and in the processing of changes in previously learned actions [16], while the anterior and posterior cingulate cortices are involved in post-feedback performance adjustments [17].

This selective minireview aims to summarize recent advances in understanding the neuromolecular underpinnings of biased cognition in depressive disorder. We begin by reviewing the core findings across two domains of biased cognitive function in depression: pessimistic judgment bias and abnormal response to negative feedback. In considering their underlying substrates, we focus on the neurochemical mechanisms identified by genetic, molecular and pharmacological challenge studies. We conclude by discussing potential experimental approaches to the treatment of depression, which are derived largely from an improved understanding of its cognitive substrates.

## 2. Pessimistic Judgment Bias

This term refers to the tendency to overestimate the likelihood of experiencing negative events while underestimating the likelihood of positive events [18]. This attitude of expecting the worst is a prominent cognitive feature of depression and can have considerable ramifications at both personal and societal levels [18,19].

The two major cognitive theories of depression, Seligman’s learned helplessness theory [20,21] and Beck’s cognitive model [22,23], emphasize the importance of hopelessness and pessimism about the future in the etiology, maintenance and treatment of depression. Moreover, according to both theories, a pessimistic view of the future and hopelessness—the belief that desirable outcomes are highly improbable and that aversive outcomes are very probable—are seen as underlying causes of depressive symptoms. Growing experimental evidence supports this notion. A study by Alloy and colleagues [24] demonstrated that depressed individuals, contrary to nondepressed controls, forecast the future for both the self and others in a pessimistic manner. In 2014, Korn and colleagues [25] demonstrated an absence of optimistic bias in belief updating in depressed individuals, and this absence correlated with symptom severity. A study by Zenger and colleagues [26] provided experimental evidence that elevated pessimism is a risk factor for anxiety and depression. Interestingly, a similar association was observed in preclinical studies in rats [27,28].

Although the theoretical rationale and experimental evidence regarding the association between pessimistic judgment bias and depression are relatively strong, information about the neuromolecular background of this interaction is still meager and requires elucidation.

One of the promising neuromolecular candidates involved in the regulation of expectations about future outcomes is dopamine (DA), a key neuromodulator in reward learning and reward-seeking behavior both in humans [29] and in animals [30]. In 2012, Sharot and colleagues [31] demonstrated for the first time that administration of a drug that enhances dopaminergic function, 3, 4-dihydroxy-L-phenylalanine (L-DOPA), impairs the ability to update belief in response to undesirable information about the future, generating optimism. Interestingly, L-DOPA was also shown to reduce pessimistic expectations by altering the value of information about potential losses, inducing, in this way, bias toward the information about potential gains [32]. Animal studies confirmed the important role of DA in the mediation of judgment bias. Thanks to the introduction of the ambiguous-cue interpretation (ACI) paradigm [33,34], a test allowing for measurement of cognitive judgment bias in animals, Rygula and colleagues demonstrated in 2014 that acute administration of the dopaminergic booster d-amphetamine produces optimism in rats [35]. This observation was confirmed several years later by Hales and colleagues [36]. Interestingly, published studies reported no optimism-inducing effects of another dopaminergic drug, cocaine [36,37]. Additionally, in 2017, Golebiowska and Rygula demonstrated, in the same animal model, that the effects of L-DOPA administration depend on the basal valence of cognitive judgment bias of experimental animals [38]. In that study, L-DOPA caused a pessimistic shift in animals classified as optimistic but had no effects on those classified as pessimistic. The inconsistency in the effects of L-DOPA between humans and animals was postulated to stem from the fact that the human study explored the valence of long-term expectations, while the animals responded to immediate rewards or punishments in the ACI paradigm. In the abovementioned study by Golebiowska and Rygula, similarly, twisted effects were observed following administration of another dopaminergic drug, the dopamine D_2_ receptor antagonist haloperidol [38]. The effects of haloperidol were reported to be opposite for animals classified as optimistic and pessimistic. The optimists became more pessimistic, while the pessimists became more optimistic. Because similar effects were observed following treatment with a serotonergic compound, the selective serotonin reuptake inhibitor (SSRI) escitalopram [38], it has been suggested that the effects of acute dopaminergic and serotonergic manipulations on pessimistic/optimistic interpretation of ambiguous cues may, at least in animals, depend on the basal valence of cognitive judgment bias [38].

The second key neurotransmitter implicated in cognitive judgment bias is serotonin (5-HT). Although in the abovementioned study by Sharot and colleagues [31], administration of the SSRI citalopram did not impact updating of beliefs about future life events, this does not imply that 5-HT function does not influence optimistic/pessimistic judgment bias. Indeed, several preclinical studies demonstrated that pharmacological modulation of 5-HT function by the administration of drugs from the family of SSRIs can change the interpretation of ambiguity in animals. In the study by Rygula and colleagues from 2014, administration of a low dose of the SSRI citalopram significantly biased animals toward the negative interpretation of the ambiguous cues in the ACI paradigm [35]. In contrast, acute administration of higher doses induced optimistic judgment bias [35]. To explain these contrasting effects, it has been proposed that the pessimistic shift observed following administration of the low dose of citalopram resulted from the temporary silencing of 5-HT system activity through stimulation of the serotonin 5-HT_1A_ autoreceptors in the raphe nuclei [39,40]. According to this hypothesis, administration of higher doses of citalopram bypasses this inhibitory mechanism, allowing for a net increase in prefrontal 5-HT levels [41,42,43,44] and optimistic shift in judgment [35]. In 2013, Anderson and colleagues demonstrated pro-optimistic effects of chronic but not acute administration of another SSRI drug—fluoxetine [45]. These results were repeated and extended by Hales and colleagues [36], who demonstrated that the pro-optimistic effects of fluoxetine on the interpretation of ambiguous cues were most pronounced after 2 and 3 weeks of treatment [36]. A study by Doyle and colleagues [46] demonstrated that administration of the 5-HT inhibitor p-chlorophenylalanine induces pessimistic-like judgment bias in sheep. A similar effect was observed following 5-HT depletion in the ACI paradigm in pigs [47].

Along with DA and 5-HT, noradrenaline (NA) is the third neurotransmitter involved in mediating optimistic and pessimistic biases. In preclinical studies using animal models, administration of drugs boosting NA neurotransmission consistently biases cognitive judgment toward pessimism. In the study by Rygula and colleagues in 2014, the NA reuptake blocker desipramine at all tested doses rendered the rats pessimistic [35]. A study testing mazindol, another noradrenergic drug with mixed noradrenergic and dopaminergic mechanism of action, reported similar results [37]. A decrease in the positive processing of the ambiguous cue in the ACI paradigm was also reported by Anderson and colleagues after acute administration of the selective noradrenaline reuptake inhibitor (SNRI) reboxetine [45]. The same drug, in combination with the stress hormone corticosterone, was reported by Enkel and colleagues to produce pessimism in their landmark study with the ACI paradigm in rats [33].

Although judgment bias was initially seen mainly as a derivative of a current affective state, which depends on the environment to which an individual is exposed, rather than a phenotypic trait, recent research unequivocally demonstrated that optimism and pessimism can be considered stable and enduring phenotypic traits [28,48,49,50,51,52]. Nonetheless, research on the genetic background of judgment bias is still scarce. In a recent study using the ACI paradigm in zebrafish, Espigares and colleagues found that telomerase-deficient fish (tert−/−) were more pessimistic in response to ambiguous stimuli than their wild-type conspecifics [53]. These telomerase-deficient mutants have shorter telomeres than their wild-type siblings and develop degenerative phenotypes characterized by, e.g., increased inflammation, which is common in aged organisms. It has been suggested that this inflammation may be responsible for the altered judgment bias. Indeed, a correlational link between pessimism and inflammation has already been reported in humans [54] and animals [48]. In the latter study, trait pessimism was reported to be associated with decreased proliferative activity of splenocytes and increased production of interleukin-(IL)1β and IL-4, activin A, l-selectin, interferon (IFN)-γ and some chemokines and receptors for advanced glycation end products [48].

While the involvement of individual genes in the development of cognitive distortions and depression has not been widely proven, there are reports of the role of 5-HT transporter (SERT) polymorphisms in the development of depression, suggesting their important role in shaping cognitive biases associated with this disorder. Indeed, individuals carrying the short allele of the SERT gene are characterized by negative cognitive bias, as they tend to display enhanced attention toward negative information and interpret ambiguous stimuli in a more pessimistic way [55,56]. Although the effects observed in humans are generally supported by studies using animal models, some studies provided mixed results. In 2014, Kloke and colleagues reported a trend for homozygous SERT knockout mice to display pessimistic bias [57]. However, this finding was not confirmed in a recent study by Krakenberg and colleagues, who did not observe interactions between the SERT genotype and biased judgment [58]. It has been suggested that, at least in mice, the association between the SERT genotype and judgment bias is not straightforward. Other factors, including multiple genes and environmental influences, are implicated in the modulation of genotype and bias.

A recent study by Boddington and collaborators suggested that individual differences in biased cognition can be partially explained by variations in brain gene expression [59]. They analyzed the expression of several dopaminergic and serotonergic genes in the prefrontal cortex of red junglefowls and reported that chicks with higher dopamine D_1_ receptor expression were more optimistic, while chicks with higher serotonin 5-HT_2A_ receptor expression tended to be less optimistic. These results further suggested the involvement of monoaminergic systems in cognitive judgment bias.

**Figure 1:**
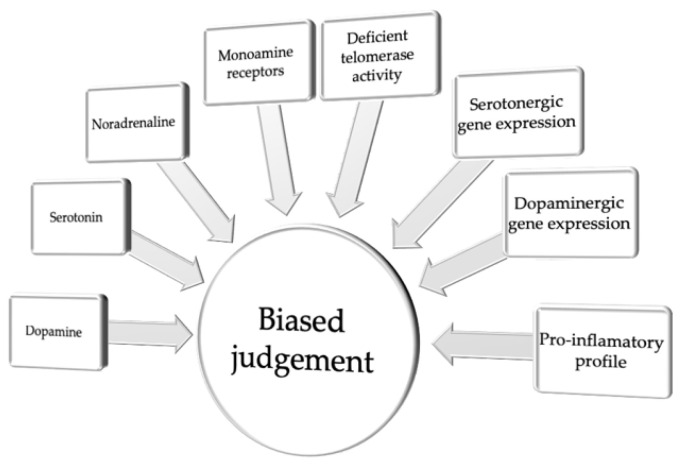
Neuromolecular and cellular correlates of biased judgment.

Taken together, although the mechanisms contributing to biased judgment in depression remain poorly understood, the majority of the studies conducted to date point to a common neuromolecular and cellular background of emotional regulation and cognitive judgment bias. This background includes various alterations in the function of the monoaminergic system function in the brain and associated physiological and cellular processes, such as a proinflammatory profile, variability in serotonergic and dopaminergic gene expression, or altered telomerase activity (Figure 1 and Table 1).

## 3. Biased Sensitivity to Feedback

People suffering from depression often ruminate over perceived failures and criticism [60]. A growing body of evidence shows that depressed individuals also have an exaggerated response to negative feedback during laboratory testing [11,61,62]. This effect was demonstrated for the first time by Elliot and colleagues [11], who found that if depressed individuals responded incorrectly on a given trial (trial N) of a simple memory task, they were disproportionately likely to fail the subsequent trial (N + 1). This “catastrophic response to perceived failure” was postulated to have an impact upon cognitive ability on any tasks that deliver performance-contingent feedback. Moreover, this effect appeared specific to depression because it was not seen in healthy controls or in any other neuropsychiatric condition [11]. The deleterious effects of hypersensitivity to negative feedback on task performance were later identified on a probabilistic reversal learning (PRL) task [61,62], during which subjects must learn to disregard misleading negative information (for review, see [63]). Apart from hypersensitivity to negative feedback, a growing number of studies examining cognitive processes in depression have suggested that depressed individuals also show hyposensitivity to positive feedback and altered processing of positively valenced information [45,64,65,66]. As postulated by Beck [23], people suffering from depression generally tend to distort environmental information negatively and thereby fail to accurately perceive or utilize positive information to modulate their behavioral responses [67,68]. Experimental evidence seems to support this notion, and numerous studies demonstrated that depressed and bipolar patients show decreased reward learning and generally reduced hedonic capacity [69,70,71].

Although studies conducted over the last two decades have shed some light on the neuromolecular background of altered sensitivity to feedback in depression, neurochemical correlates are still far from fully understood. One of the natural and most frequently studied neurotransmitters in this context is DA. In an already classic study from 2004, Frank and colleagues demonstrated that decreased availability of DA, which can be observed, e.g., in Parkinson’s patients off medication, is associated with better learning from negative feedback than from positive feedback [72]. Importantly, DA medication reversed this bias, making patients more sensitive to positive than negative outcomes. The computational model of reinforcement learning applied in the study allowed for the prediction of the abovementioned effects [72]. In a model proposed one year later, Frank postulated that low DA availability shifts the neurochemical balance in the basal ganglia toward an indirect (“NoGo”) pathway and impairs learning from positive feedback in comparison to learning from punishment [73]. In contrast, high DA availability leads to direct (“Go”) pathway overactivity and improves learning from positive feedback compared to learning from negative feedback [73]. A growing number of pharmacological, neuroimaging and genetic studies seem to support this model. Pharmacological studies have demonstrated that modulation of dopamine D_2_ receptors affects learning only from positive feedback but not from negative feedback [29,70,71,74], suggesting their specific involvement in learning from a reward. However, other studies in humans [75,76] and in animals [77,78,79,80] pointed to the specific role of dopamine D_2_ receptors in avoiding negative outcomes [73,81]. A recent study by Lim and collaborators [82] confirmed the blunting effects of dopamine D_2_ receptor agonism on learning from negative feedback in healthy participants and reported similar effects on learning from punishment, following the administration of the dopamine D_2/3_ receptor antagonist amisulpride. Such a nonselective effect of dopamine D_2/3_ receptor antagonism has already been previously reported [83,84], suggesting that these receptors are generally involved in feedback-based learning. Indeed, an elegant study by Cox and colleagues [76] demonstrated, using positron-emission tomography (PET), that individual differences in dopamine D_1_ and D_2_ receptor binding predict the effectiveness of learning from positive and negative feedback, respectively, and that DA depletion improves learning from negative feedback via dopamine D_2_ receptor signaling.

As proposed by Frank and O’reilley [81] and supported by the abovementioned studies, higher levels of DA (e.g., during unexpected rewards) switch the balance within the DA nigro-striatal circuit towards higher sensitivity of dopamine D_1_ receptors to its endogenous ligand, leading to activation of the (direct) “Go” pathway. On the contrary, DA depletion (e.g., during lack of reward) switches the balance toward higher sensitivity of dopamine D_2_ receptors and leads to activation of the (indirect) “No-Go” pathway. Therefore, activation of dopamine D_1_ receptors improves learning from positive feedback, while activation of dopamine D_2_ receptors improves learning from negative feedback.

Research by Cools and collaborators [85] revealed that subjects with high basal DA synthesis in the striatum show relatively better reversal learning from unexpected rewards than from unexpected punishments, whereas subjects with a low basal level of DA synthesis show the reverse pattern. In 2007, Frank and colleagues [86] demonstrated that three polymorphisms in genes associated with DA function contribute to reward and avoidance learning in humans. A polymorphism in the DARPP-32 gene (which encodes for a dopamine- and cAMP-regulated phosphoprotein that is a crucial mediator of the biochemical effects of DA) predicted relatively better probabilistic reward learning; the C957T polymorphism of the dopamine D_2_ receptor gene, associated with striatal dopamine D_2_ receptor function, predicted the degree to which participants learned to avoid choices that had been probabilistically associated with negative outcomes; and the Val/Met polymorphism of the catechol-O-methyltransferase (COMT) gene, associated with prefrontal cortical DA function, predicted participants’ ability to rapidly adapt behavior on a trial-to-trial basis. A specific role of another dopamine D_2_ receptor gene polymorphism (DRD2-TAQ-IA) in feedback-based learning in a human neuroimaging paradigm was demonstrated in 2007 by Klein and colleagues [87] and in 2009 by Jocham and colleagues [88]. In the former, A1 allele carriers with lower dopamine D_2_ receptor densities learned to avoid actions with negative consequences less efficiently than those without the allele. In the latter, the A1 subjects demonstrated an impaired ability to sustain a newly rewarded response after a reversal of the stimulus-reward contingency in the PRL task and showed a generally decreased tendency to stick with a rewarded response.

A growing number of studies have also implicated 5-HT in the modulation of sensitivity to feedback. Published reports suggest that increasing 5-HT transmission leads to a reduced sensitivity to aversive outcomes, whereas decreasing 5-HT transmission, by way of either acute tryptophan depletion, presynaptic receptor stimulation or upregulation of SERT, leads to an increased sensitivity to aversive outcomes (reviewed by [63]). In the pioneering study by Chamberlain and colleagues [89], a low, acute dose of the SSRI citalopram, which was postulated to activate presynaptic 5-HT autoreceptors and in this way downregulate 5-HT transmission, increased the tendency to switch the response following misleading negative feedback in the PRL task, mimicking the increased sensitivity to negative feedback observed in depression [61]. This effect of acute SSRI treatment was replicated 12 years later by Skandali and colleagues using escitalopram [90]. Similar effects of reduced 5-HT function were reported following acute tryptophan depletion, a procedure that has been used extensively to study the effect of low 5-HT levels in humans [91,92]. Additionally, a complex report by den Ouden and collaborators [93] revealed that allelic variation in SERT predicts negative feedback sensitivity (behavioral adaptation following punishment). Specifically, L′ homozygosity, which has been linked with increased SERT binding and decreased levels of extracellular 5-HT [94], was associated with increased sensitivity to negative feedback [93]. Studies in humans are complemented by results from animal models. In 2010, Bari and colleagues [95] repeated the effect observed by Chamberlain and collaborators in humans [89] using a pre-clinical version of the PRL test in rats treated with citalopram, while the study by Ineichen and colleagues demonstrated that in mice, a genetic reduction in SERT function leads to reduced sensitivity to negative feedback [96]. In 2015, Rygula and colleagues showed that selective 5-HT depletion in the amygdala increases sensitivity to aversive feedback and reduces punishment-induced response suppression in nonhuman primates [97], and a study by Phillips and colleagues revealed that sensitivity to positive feedback can be modulated by pharmacological targeting of serotonin receptor 5-HT_2C_ [98]. In the latter study, administration of the serotonin 5-HT_2C_ receptor antagonist SB 242084 reduced sensitivity to positive feedback, while administration of the serotonin 5-HT_2C_ receptor agonist WAY 163909 resulted in changes associated with increased sensitivity to positive feedback and decreased sensitivity to negative feedback. It has been proposed that oversensitivity to negative feedback associated with low levels of tonic 5-HT could represent either enhanced prediction error signals, brought about by an increased signal-to-noise ratio of phasic 5-HT bursts [99], or attenuated punishment-induced response suppression, which can be defined as an instrumental process that inhibits behavior by virtue of the link between responses and the aversive outcomes they produce and a Pavlovian process that reflexively suppresses behavior [100].

In 2017, Rychlik and colleagues reported the role of glutamatergic neurotransmission in mediating sensitivity to feedback [101]. In this study, acute treatment with the prototypic, fast-acting antidepressant, N-methyl-D-aspartate (NMDA) receptor antagonist ketamine, significantly and persistently diminished the sensitivity of rats to negative feedback in the preclinical version of the PRL paradigm in a manner similar to that observed following the administration of higher doses of the SSRI citalopram [95].

Recent research with a pharmacogenetic model of reduced neurogenesis and a translationally relevant PRL paradigm demonstrated novel functions for adult-born neurons in sensitivity to rewards and negative feedback [102]. In that study, transgenic male rats that lacked adult neurogenesis were impaired in the use of probabilistic reward feedback to guide choice toward more profitable options. The observed effect was speculated to be due to either the specific loss of newborn neurons or to downstream changes that may have arisen over several weeks of neurogenesis ablation and pointed at hippocampal function in the modulation of sensitivity to feedback [102].

A study by Vaselic and colleagues revealed that reward processing and learning can be influenced by the sex hormone estradiol, which increases sensitivity to positive feedback and that these effects can be partially modulated by striatal DA transporter (DAT1) genes and personality traits related to reward sensitivity [103]. Similar feedback modulating effects were observed following acute administration of the neuropeptide oxytocin, which acutely increased the sensitivity of rats to positive feedback [104].

The studies by Bryce and Floresco [105] and Dieterich [106] revealed the role of stress hormones in sensitivity to feedback. In the former, increased corticotropin releasing factor (CRF) signaling reduced negative feedback sensitivity in rats, while in the latter, chronic corticosterone administration decreased the sensitivity of mice to positive feedback, which has been interpreted as a robust blunting of positive processing [106].

**Figure 2:**
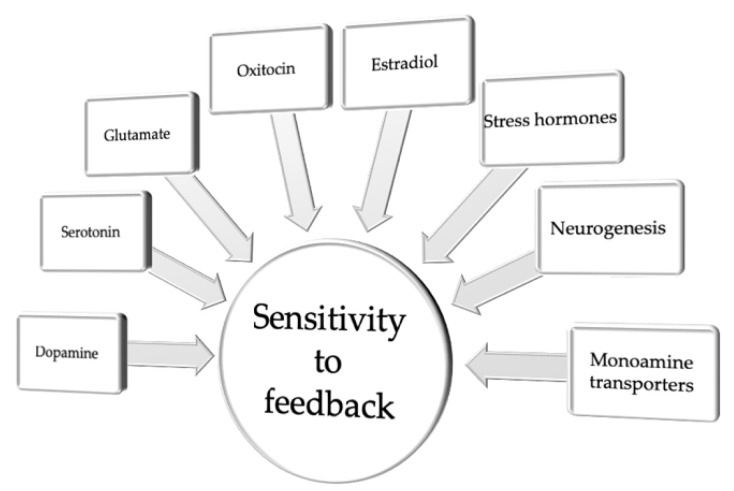
Neuromolecular and cellular correlates of sensitivity to feedback.

To summarize this section, key components of the neuromolecular puzzle constituting sensitivity to feedback include components of the dopaminergic, serotonergic and glutamatergic neurotransmitter systems, as well as sex and stress hormones and alterations in neurogenesis (Figure 2 and Table 1).

## 4. Implications for Treatment

Contemporary pharmacological treatment strategies for depressive disorder are directed at drugs that block the reuptake of 5-HT (the SSRIs) and/or NA (the SNRIs) from the extracellular space, which is thought to enhance the neural activity of these systems over time. Although clinical evidence clearly shows that the beneficial effects of these drugs occur with prolonged (min. 4–6 weeks) treatment, this delay in antidepressant action seems to be counterintuitive, as the molecular, cellular and chemical effects occur very quickly after a drug is administered, and recent studies have demonstrated that beneficial effects on biased cognition are evident even after the first dose. The cognitive neuropsychological theory of antidepressant action [107,108] was developed in an attempt to understand this potential paradox and states that the induction of a more positive way of processing environmental stimuli (positive bias) leads to cognitive and psychological reconsolidation [107]. This theory is consistent with cognitive models of depression, which emphasize the importance of correcting negative biases in information processing in the successful treatment of this disorder [23,109]. Indeed, there is now a growing body of experimental evidence indicating that by targeting cognitive biases, antidepressants can affect emotional processing very early in treatment and independently of changes in subjective mood (reviewed by Serra and colleagues [110]). Studies in animals confirmed that acute administration of several widely prescribed antidepressants changes cognitive judgment bias in the ACI test (citalopram [35], desipramine [35] and reboxetine [36]) and the sensitivity of rats to performance feedback in the preclinical version of the PRL task (citalopram [95] and ketamine [101]). Monitoring this positive shift in emotional processing creates an opportunity for fast detection of agents with antidepressant potential. This could be applied to new molecules in the early phase of development or the repurposing of existing drugs. As mentioned by Godlewska and Harmer [111], the use of this simple measure could also allow relatively inexpensive screening of the best treatment regimen by testing different doses or treatment periods in smaller groups of individuals before running large clinical trials. Another emerging opportunity associated with studying the effects of antidepressant drugs on cognitive biases is to explain the mode of action of new fast-acting antidepressants, such as the NMDA receptor antagonist ketamine, which acts on mood within hours. It has been proposed that the impact on emotional processing by this group of drugs is different than that of conventional antidepressant drugs and that they might act by blocking the retrieval of negative memory associations. This effect could also modulate sensitivity to negative feedback [101].

## 5. Conclusions and Future Directions

The aforementioned advances in studying neuromolecular correlates of biased cognition in depression are both exciting and timely. We are sure that the next few years will see exciting discoveries emerge from a new focus on this level of inquiry. This progress must evolve from the collection of experimental data from various brain areas and neural networks through the integration of a biologically grounded theoretical framework with deeply analogous animal research using the newest neuromolecular techniques to human cognitive neuroscience and clinical psychiatry. Indeed, current single-cell genomic technology already allows us to obtain new molecular mechanistic insights from the brains of depressed patients. Other emerging techniques include transcranial focused ultrasound/radiation methods. These approaches can be improved by combining them with the molecular mechanisms suggested in our manuscript. It seems also crucial to combine the levels of inquiry tackling at the same time molecular mechanisms and whole circuits. When seeking a better understanding of the neuromolecular background of cognitive biases in depression, we need to actively commit to synergy.

## Figures and Tables

**Table 1:** Pharmacological manipulations targeting various neurotransmitter systems, which affect pessimistic judgment bias or sensitivity to feedback.

Pessimistic Judgment Bias					
Target System	Drug Used	Study Subject	Test	Behavioral Outcome	Reference
DA	L-DOPA	Human	BUT	Impaired ability to update belief in response to undesirable information about the future, higher optimism	[31]
DA	L-DOPA	Human	SMT	Shift bias toward the information about gains	[32]
DA	d-amph	Rat	ACI	Optimism	[35]
DA	L-DOPA	Rat	ACI	Pessimistic shift in animals classified as optimistic	[38]
DA	Halo	Rat	ACI	Optimists became more pessimistic, while pessimists became more optimistic	[38]
5-HT	Escit	Rat	ACI	Pessimistic shift in animals classified as optimistic	[38]
5-HT	Cit	Human	BUT	No effect	[31]
5-HT	Cit	Rat	ACI	Negative interpretation of ambiguous cues (a low dose) or optimistic judgment bias (a high dose)	[35]
5-HT	Flx	Rat	ATDT	Pro-optimistic effects of chronic treatment	[45]
5-HT	Flx	Rat	ACI	Pro-optimistic effects of chronic treatment	[36]
5-HT	pCPA	Sheep	SDT	Pessimistic judgment bias	[46]
5-HT	pCPA	Pig	ACI	Pessimistic judgment bias	[47]
NA	Desi	Rat	ACI	Pessimistic judgment bias	[35]
NA/DA	Mazin	Rat	ACI	Pessimistic judgment bias	[37]
NA	Rbx	Rat	ACI	Decrease in the positive processing	[45]
NA	Rbx, Cort	Rat	ACI	Pessimistic judgment bias	[33]
**Sensitivity to feedback**					
**Target System**	**Drug Used**	**Study Subject**	**Test**	**Behavioral Outcome**	**Reference**
DA	L-DOPA	Human	PLT	Higher sensitivity to positive than negative outcomes in PD patients on medication	[72]
DA	L-DOPA, Halo	Human	G/NG	Subjects treated with L-DOPA have a greater propensity to choose the most rewarding action relative to subjects treated with haloperidol	[29]
DA	Sulp	Human	RLT	Impairment in reward choice performance	[74]
DA	APTD	Human	PST	Improved learning from negative outcomes	[76]
DA	Quin	Rat	SPRL	Impaired learning from negative feedback	[79]
DA	Raclo, Quin	Rat	PRL	Negative feedback learning depends on D_2_R signaling, whereas learning from positive feedback depends on D_1_R signaling	[80]
DA	Halo	Human	PST	Increased DA release during positive feedback enhanced Go learning for good choices	[81]
DA	Ami, Prami	Human	RL	Impaired learning from negative feedback	[82]
5-HT	Cit	Human	PRL	Low dose increased tendency to switch the response following negative feedback	[89]
5-HT	Escit	Human	PRL	Impaired learning with uncertain reinforcement and enhanced responsivity to negative feedback	[90]
5-HT	ATD	Human	PRL	Increased punishment prediction	[92]
5-HT	SB 242084	Mice	PRL	Reduced sensitivity to positive feedback	[98]
5-HT	WAY 163909	Mice	PRL	Increased sensitivity to positive feedback and decreased sensitivity to negative feedback	[98]
glu	Ket	Rat	PRL	Diminished the sensitivity of rats to negative feedback	[101]

5-HT—serotonin, ACI—the ambiguous interpretation test, Ami—amisulpride, APTD—acute phenylalanine and tyrosine depletion, ATD—acute tryptophan depletion, ATDT—the affective tone discrimination task, d-amph—d-amphetamine, BUT—the belief updating test, Cit—citalopram, Cort—corticosterone, D1R—dopamine D1 receptor, D2R—dopamine D2 receptor, DA—dopamine, Desi—desipramine, Escit—escitalopram, Flx—fluoxetine, glu—glutamate, G/NG—Go/NoGo task, Halo—haloperidol, Ket—ketamine, L-DOPA—3,4-dihydroxy-L-phenylalanine, Mazin—maziodol, NA—noradrenaline, pCPA—p-Chlorophenylalanine, PD—Parkinson’s disease, PLT—the procedural learning task, Prami—pramipeskole, PRL—the probabilistic reversal learning test, PST—the probabilistic selection task, Quin—quinpirole, Raclo—raclopride, Rbx—reboxetine, RL—reversal learning taks, RLT—the reinforcement learning task, SB 242084—selective antagonist of the 5-HT2C receptor, SDT—the spatial differentiation task, SMT—the stock market task, SPRL—the spatial reversal learning test, Sulp—sulpiride, WAY 163909—selective agonist for the serotonin 5-HT2C receptor.

## Data Availability

No new data were created or analyzed in this study. Data sharing is not applicable to this article.
